# Quality assessment of systematic reviews or meta-analyses of nursing interventions conducted by Korean reviewers

**DOI:** 10.1186/1471-2288-12-129

**Published:** 2012-08-28

**Authors:** Hyun-Ju Seo, Kyeong Uoon Kim

**Affiliations:** 1 Department of Nursing, College of Medicine, Chosun University, 309 Pilmum-daero, Dong-gu Gwangju, 501-759, Republic of Korea; 2 Department of Nursing, College of Oriental Medicine, Public Health & Welfare, Samcheok 2nd Campus, Kangwon National University, 3 Hwangjo-ri, Dogye-eup, Samcheok-si, Kangwon-do 245-907, Republic of Korea

**Keywords:** Systematic review, Meta analysis, Quality assessment, Nursing intervention

## Abstract

**Background:**

A systematic review is used to investigate the best available evidence of clinical safety and effectiveness of healthcare intervention. This requires methodological rigor in order to minimize bias and random error. The purpose of this study is to assess the quality of systematic reviews or meta-analyses for nursing interventions conducted by Korean researchers.

**Methods:**

We searched electronic databases from 1950 to July 2010, including ovidMEDLINE, ovidEMBASE, and Korean databases, including KoreaMed, Korean Medical Database, and Korean studies Information Service System etc. Two reviewers independently screened and selected all references, and assessed the quality of systematic reviews or meta-analyses using the “Assessment of Multiple Systematic Reviews" (AMSTAR) tool.

**Results:**

Twenty two systematic reviews or meta-analyses were included in this study. The median overall score (out of 11) for included reviews was 5 (range 2–11) and the mean overall score for AMSTAR was 4.7 (95% confidence interval 3.8-5.7). Nine out of 22 reviews were rated as low quality (AMSTAR score 0–4), 11 were rated as moderate quality (AMSTAR score 5–8), and two reviews were categorized as high quality (AMSTAR score 9–11).

**Conclusions:**

The methodological quality of published reviews on nursing interventions conducted by Korean reviewers was assessed as low to moderate. In order to use the best available evidence in clinical decision making, reviewers should conduct systematic reviews or meta- analyses using rigorous research methods.

## Background

Due to regulations of new health technology, reimbursement of new drugs, and dissemination and implementation of clinical practice guidelines, the use of scientific evidence in clinical and health policy decision making has been increasing. In recent times, evidence-based nursing practice has received the attention of clinical nurses in South Korea. Evidence-based nursing is a process of implementing clinical decision making based on clinical expertise, patient preference, and the best available evidence using accessible resources [1].

A systematic review is used to investigate the best available evidence of clinical safety and effectiveness of healthcare interventions and is conducted using a systematic approach to minimize bias and random error described in research subjects and methods [2]. In addition, systematic reviews or meta-analyses were conducted in order to facilitate development of clinical practice guidelines for professionals and to plan and conduct of new researches [3,4]. Therefore, a high-quality, well-designed systematic reviews should be conducted and published in order to provide the best available evidence to healthcare providers. The reason for assessing the quality of systematic reviews is to examine the confidence of review findings. Quality can be defined as ‘the extent of standards to protect all aspects of research design and implementation from systematic error, unsystematic error, and inferential error.’ Quality assessment is also important for judging the overall strength of evidence on given research questions and is considered in evaluation of the methodological quality of a research design and implementation rather than the intervention’s true effect in the process of research [5].

Several reviews have been conducted for assessment of the methodological quality of systematic reviews of pharmaceutical care [3], oral health care [6], dental healthcare [7], pediatric oncology [8], and traditional Chinese medicine interventions [9]. Using the AMSTAR tool, three out of four systematic reviews on oral healthcare interventions published in the Journal of Applied Oral Science were rated as low quality [6]; a critical appraisal of 110 systematic reviews in orthodontics were found to be of moderate quality and no significant trend toward quality improvement was observed [7]. More than half of 31 included systematic reviews were assessed as moderate quality using AMSTAR in pharmacist health intervention [3]. Using the Overview Quality Assessment Questionnaire, most of 117 systematic reviews in the field of pediatric oncology were found to have serious methodological flaws undermining evidence-based decision making [8]. Compliance of systematic reviews of traditional Chinese medicine published in Chinese journals using AMSTAR checklist items varied from 0 ~ 70.2%, concluding that the quality of 396 systematic reviews was serious [9].

There was a lack of information on the methodological quality of systematic reviews or meta-analyses performed by Korean reviewers. The aim of this study was 1) to assess the methodological quality of systematic reviews or meta-analyses related to nursing interventions published by Korean authors, and 2) to identify methodological quality domain rated as improperly performed.

## Methods

### Inclusion criteria

We included systematic reviews or meta-analyses of the treatment effects on nursing interventions published in English or Korean by Korean reviewers. There was no limitation on the type of intervention, clinical setting, and study population.

### Search strategy

For retrieval of systematic reviews or meta-analyses, a search of Ovid-Medline and Ovid-Embase was conducted on July 1 ~ 3, 2010, and Korean databases, including the Korean studies Information Service System, Korea Institute of Science and Technology Information, Korean Medical Database, KoreaMed, and National Discovery for Science Leaders were searched on July 13 ~ 14, 2010.

In the electronic databases, we used search words (nurs*.mp. or "nurs*".jn.) to find journals or primary studies related to nursing science, and combined words related to Korea (exp Korea/or Korea$.mp. or Korea$.ia. or Korea$.cp.) to search studies conducted by Korean authors. We then increased specificity of the search using search filters for systematic reviews or meta-analyses [10]. “Systematic review” or “meta-analysis” was used for all Korean databases, and “meta bunseok,” “chegyejeok munheon,” or “chegyejeok munheon gochal” was used in the searchable Korean databases. Besides, “Ganho” was added when conducting a search within the Korea Institute of Science and Technology Information, and “systematic review” or “meta-analysis” and nurs* were combined in KoreaMed for improvement of specificity ( Additional file 1).

### Study selection and data extraction

**Figure 1:**
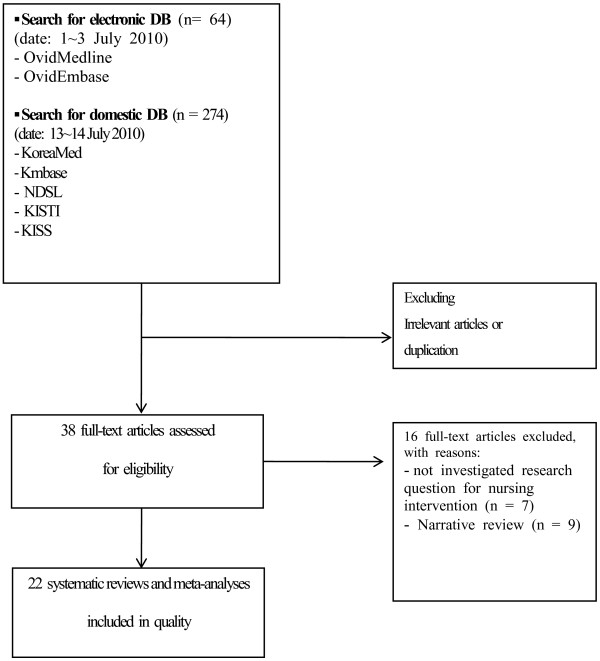
Flow diagram of the review selection.

Screening of titles and abstracts of retrieved records for inclusion criteria was performed by two independent reviewers. The full texts of eligible reviews were obtained and examined to determine whether they met the inclusion criteria. Any disagreements were resolved by consensus through discussion (Figure 1).

### Quality assessment

We evaluated the methodological quality of meta-analyses or systematic reviews using Assessment of Multiple Systematic reviews (AMSTAR) [11]. Eleven items were rated as “Yes”, “No”, “Can’t answer”, or “Not applicable.” AMSTAR, developed in 2007, has substantial inter-rater agreement of the individual items with a mean kappa of 0.70 (95% confidence interval: 0.57, 0.83). The Intra Class Correlation Coefficient of the total score for AMSTAR was 0.84 (95% confidence interval: 0.65, 0.92), compared with 0.91 (95% confidence interval: 0.82, 0.96) for the Overview Quality Assessment Questionnaire, and 0.86 (95% confidence interval: 0.71, 0.94) for the Sacks’ instrument. In testing feasibility, AMSTAR was evaluated as easy to apply, in terms of the time required for completion of an assessment: approximately 10–15 minutes [12]. In external validation, the reliability of the total AMSTAR score between two reviewers (the sum of all items answered ‘Yes’ scored as 1, all others as 0) was almost perfect (kappa 0.8; 95% confidence interval: 0.67 to 1.00) and Pearson’s R was 0.96 (95% confidence interval: 0.92 to 0.98). Inter-rater agreement (kappa) between two assessors for the global assessment was substantial (kappa 0.63; 95% confidence interval: 0.40, 0.88) [13].

In this study, the total score for AMSTAR was calculated by summing one point for each “yes” and no point for others, including “no”, “can’t answer”, and “not applicable,” resulting in summary scores from 0 to 11. In order to rate the quality of the reviews, we have applied the following three categories: a score of 0–4 is classified as low quality, 5–8 indicates moderate quality, and 9–11 is regarded as high quality [14]. Quality assessment was performed independently by two authors, and discrepancies were discussed until an agreement was reached.

### Data analysis

For each included review, we presented the tabulation of scoring for each AMSTAR domain (Yes, No, Can’t answer, Not applicable). Data were summarized descriptively as median/mean and 95% confidence interval of total AMSTAR score. The time trend of the number and quality of included reviews was examined. We showed the number of reviews categorized as high, moderate, and low quality according to the pre-specified definition.

## Results

### Review selection

A flowchart of the review selection is shown in Figure 1. The initial search yielded 338 records of potential interest; of these, 300 were excluded based on the title or abstract. The remaining 38 reviews were obtained and read in their full text. Of the 38 remaining reviews, 16 reviews were excluded from the study for the following reasons: seven reviews were not research on nursing interventions and nine reviews were narrative reviews ( Additional file 2). The remaining 22 reviews were included in the quality assessment ( Additional file 3). All reviews were systematic reviews and meta-analyses on nursing interventions conducted by Korean reviewers.

### Review characteristics

Characteristics of the 22 systematic reviews and meta-analyses are as follows ( Additional file 4): the reviews were reported between 1992 and 2009. The number of studies included in meta-analysis ranged from 5 to 64 intervention studies. Ten reviews were performed on relatively homogeneous patients or populations; however, more than half of the studies included diverse participants, from healthy subjects to patients with chronic disease. There were five reviews on the effectiveness of relaxation therapy, three reviews on exercise intervention, and two reviews of smoking cessation programs. Most of the systematic reviews and meta-analyses investigated multiple interventions for participants with various conditions rather than focused research questions. Regarding study design, most studies included in meta-analyses or systematic reviews were randomized controlled trials or quasi-experimental studies including a pre and post design.

### Methodological quality

The assessment of methodological quality is described in Table 1. The median overall score (out of 11) for all 22 included reviews was 5 (range 2–11), and mean overall score of AMSTAR was 4.7 (95% confidence interval 3.8-5.7). Table 2 shows results of the methodological quality assessment according to each item. Three reviews (13.6%) were performed in duplicate study selection and data extraction. The status of publications such as gray literature searching was used as an inclusion criterion in four reviews (18.2%). Fourteen reviews (63.6%) did not provide information on both included and excluded studies.

**Table 1:** Methodological Quality of systematic reviews or meta-analyses using AMSTAR

**Reference number**	**Publication year**	**Quality Items**											**AMSTAR score**
		**1**	**2**	**3**	**4**	**5**	**6**	**7**	**8**	**9**	**10**	**11**	
1	1992	Y	CA	Y	CA	N	Y	CA	Y	Y	N	N	5
2	1996	Y	Y	CA	CA	Y	Y	N	N	N	N	Y	5
3	1998	Y	N	N	N	Y	Y	N	N	N	N	N	3
4	1998	Y	N	CA	CA	N	Y	CA	N	Y	Y	Y	5
5	2000	Y	N	N	N	N	Y	N	N	N	N	N	2
6	2000	Y	N	Y	CA	N	Y	N	N	Y	N	Y	5
7	2001	Y	N	CA	N	N	Y	N	N	N	N	N	2
8	2001	Y	N	N	CA	CA	Y	N	N	Y	N	Y	4
9	2002	Y	N	N	CA	N	Y	N	N	Y	N	N	3
10	2003	Y	N	Y	CA	N	Y	N	N	Y	N	Y	5
11	2003	Y	N	CA	N	N	Y	N	N	N	N	N	4
12	2003	Y	N	CA	CA	N	Y	N	N	Y	N	Y	3
13	2003	Y	N	CA	CA	N	Y	N	N	Y	N	N	2
14	2004	Y	N	Y	CA	CA	Y	N	N	Y	N	Y	5
15	2004	Y	N	N	CA	CA	Y	N	N	Y	Y	N	4
16	2005	Y	CA	Y	N	N	Y	N	N	Y	N	Y	5
17	2007	Y	N	Y	Y	N	Y	N	N	N	N	Y	5
18	2007	Y	Y	Y	Y	Y	Y	Y	Y	Y	Y	Y	11
19	2008	Y	N	Y	CA	N	Y	N	N	Y	Y	Y	6
20	2009	Y	N	Y	N	N	Y	N	N	Y	Y	N	5
21	2009	Y	N	Y	Y	CA	Y	Y	CA	Y	N	N	6
22	2009	Y	Y	Y	Y	Y	Y	Y	Y	Y	CA	N	9

Y: Yes, N: No, CA: Can’t answer, NA: Not applicable.

**Table 2:** AMSTAR Assessment according to quality items

**Quality items**		**“Yes” (%)**	**“No”**	**“Cannot answer”**	**“Not applicable”**
		**n (%)**	**n (%)**	**n (%)**	**n (%)**
1	Was an "a prior" design provided?	22 (100.0)	0	0	0
2	Was there duplicate study selection and data extraction?	3 (13.6)	17 (77.3)	2 (9.1)	0
3	Was a comprehensive literature search performed?	11 (50.0)	5 (22.7)	6 (27.3)	0
4	Was the status of publication (i.e. grey literature) used as an inclusion criterion?	4 (18.2)	6 (27.3)	12 (54.5)	0
5	Was a list of studies (included and excluded) provided?	4 (18.2)	14 (63.6)	4 (18.2)	0
6	Were the characteristics of the included studies provided?	22 (100.0)	0	0	0
7	Was the scientific quality of the included studies assessed and documented?	3 (13.6)	17 (77.3)	2 (9.1)	0
8	Was the scientific quality of the included studies used appropriately in formulating conclusions?	3 (13.6)	18 (81.8)	1 (4.5)	0
9	Were the methods used to combine the findings of studies appropriate?	16 (72.7)	6 (27.3)	0	0
10	Was the likelihood of publication bias assessed?	5 (22.7)	16 (72.7)	1 (4.5)	0
11	Was the conflict of interest stated?	11 (50.0)	11 (50.0)	0	0

Three reviews (13.6%) assessed and documented the quality of the included studies and drew an appropriate conclusion reflecting the scientific quality of the included studies, and 16 reviews (72.7%) appropriately combined the findings of studies using meta-analytic methods. For the 22 evaluated reviews, nine were rated as low quality (AMSTAR score 0–4), 11 were found to be of moderate quality (AMSTAR score 5–8), and two reviews were categorized as high quality (AMSTAR score 9–11).

**Figure 2:**
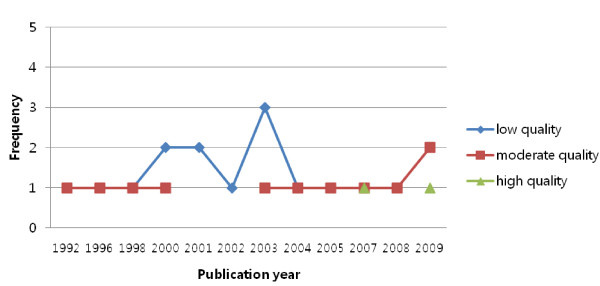
The time trend of the number and quality of included reviews.

In addition, no time trend was observed with regard to the number and quality of systematic reviews or meta-analyses published from 1992 to 2009 (Figure 2).

## Discussion

To the best of our knowledge, this is the first study to examine the quality of systematic reviews or meta-analyses on the effectiveness of nursing interventions published by Korean researchers using AMSTAR. We found that most of the reviews were of low to moderate quality. Only two recently published reviews were found to be of high quality, whereas half of the 22 reviews were rated as moderate quality, and 41% of reviews were rated as low quality. Therefore, we might conclude that the pooled effect estimates reported in systematic reviews or meta-analyses of nursing interventions were susceptible to methodological flaws, which seriously weaken confidence in the estimated treatment effect. These findings were similar to those of a previous study by reported Shin et al. [15]; among a total of 209 articles published in the Journal of the Korean Academy of Nursing between 2007 and 2008, none of the studies were evaluated as having a high level of evidence (graded as 1++) and 1.0% of the studies were rated as 1+ based on Scottish Intercollegiate Guidelines Network quality criteria. Several studies have reported empirical evidence indicating that studies with methodological flaws may report more beneficial effects.

In our study, 13.6% of the included reviews appropriately conducted the quality assessment of the primary studies, and three of the 22 reviews linked quality to the interpretation of results. This finding is consistent with studies suggesting that 88.5% of systematic reviews carried out quality assessment, 51.4% reflected the results of evaluation [16], 45% of reviews assessed and documented the scientific quality of the included studies, and 42% of reviews adequately concluded accounting for the methodological quality [3].

In this study, use of the publication status as selection criteria and documentation of a list of studies included and excluded from the review accounted for only 18.2% of reviews. In assessing traditional Chinese medicine interventions, less than 5% of 369 reviews were compliant with this item [9]. Papageorgiou et al. reported that approximately one forth of 110 reviews used the publication status as inclusion criteria and reported a list of included and excluded studies [7].

Selection of studies and data extraction should be performed by at least two independent reviewers and a consensus should be reached for disagreements in order to increase transparency and reproducibility of systematic review or meta-analysis. In this study, this item was performed appropriately in only 13.6% of included reviews. In a study evaluating the quality of 117 systematic reviews in pediatric oncology, performance of duplicate study selection and data extraction was reported in 19.7% of the reviews [8]. Two of four reviews of oral healthcare interventions published in the Journal of applied oral science [6], 41.2% of 369 systematic reviews of traditional Chinese medicine interventions [9], 41.8% of 110 systematic reviews in orthodontics [7], and 58 % of 31 systematic reviews on pharmacist health interventions were adequately performed [3].

Evidence-based practice involves the use of research evidence in clinical decision making. Therefore, a well-designed systematic review or meta-analysis should be performed in order to provide healthcare providers with the best available evidence. When conducting systematic reviews or meta-analyses on the effectiveness of nursing interventions, systematic reviewers and meta-analysts should keep in mind the quality items based on the AMSTAR instrument.

We found that 72.3% of studies were rated as “cannot answer” for at least one item. The answer “cannot answer” means that an item is relevant but is not described by the authors. This may indicate inadequate reporting of a systematic review or meta-analysis. Although the quality of reporting has shown improvement in recently published studies, there is still room for improvement to ensure transparency and reproducibility. The PRISMA group has recommended the use of the PRISMA checklist to improve reporting of systematic reviews and meta-analyses [17,18].

Our study has some limitations. First, this study included systematic reviews or meta-analyses of nursing interventions published in Korean or English by Korean researchers. Given that only 22 reviews were included in the evaluation, our results have a limitation on generalisability of data. Second, there are several factors affecting the quality of reviews, including the areas of healthcare, language, and clinical expertise of reviewers. However, we only examined the relationship between year of publication and quality. Third, we did not compare our results to reviews conducted in other areas of healthcare or other countries.

Future research is needed to investigate the strategies to improve the methodological quality and reporting quality of systematic reviews or meta-analyses of the effectiveness of nursing interventions.

## Conclusions

This critical appraisal of systematic reviews or meta-analyses in the field of nursing interventions published by Korean researchers illustrated that most of 22 reviews is low-to-moderate quality using the AMSTAR tool. Therefore, reviewers should focus on improving the quality of systematic reviews or meta-analyses, rather than continuing to publish in quantity.

Systematic reviews or meta-analyses with high quality aid healthcare providers in keeping their knowledge up-to-date and provide the best available evidence for clinical decision-making in regard to the benefit and harm of nursing interventions. Therefore, reviewers should perform systematic reviews or meta-analyses based on rigorous research methods.

## Competing interests

The authors have no conflict of interest.

## Authors’ contributions

SHJ wrote the manuscript and analyzed the data. KKU designed and conducted the study and commented on the earlier drafts. SHJ and KKU participated in assessing the quality of included reviews. Both authors contributed towards the conception and design of the study and read and approved the final manuscript.

## Pre-publication history

The pre-publication history for this paper can be accessed here:

http://www.biomedcentral.com/1471-2288/12/129/prepub

## Supplementary Material

Additional file 1 **Appendix 1. **Search strategies. Click here for file

Additional file 2 **Appendix 2. **Reasons and a list of excluded reviews. Click here for file

Additional file 3 **Appendix 3. **A list of included reviews. Click here for file

Additional file 4 **Appendix 4. **Characteristics of reviews included. Click here for file
