# Supplementary material for: Quality assessment of systematic reviews or meta-analyses of nursing interventions conducted by Korean reviewers
Source: BMC Med Res Methodol. 2012 Aug 28;12:129. doi: 10.1186/1471-2288-12-129 (PMC3552770; doi:10.1186/1471-2288-12-129)
Supplement: Additional file 1 — Appendix 1. Search strategies. [file 1471-2288-12-129-S1.doc]

**Appendix 1 Search strategies**

| **Search strategy for ovidMedline** | |  |
| --- | --- | --- |
| 1 | nurs*.mp. | 92,723 |
| 2 | exp Korea/ or exp South Korea/ | 11,589 |
| 3 | korea$.in. | 115,979 |
| 4 | korea$.cp. | 16,738 |
| 5 | korea$.mp. | 24,079 |
| 6 | 2 or 3 or 4 or 5 | 122,359 |
| 7 | nurs*.jn. | 3,600 |
| 8 | 1 or 7 | 93,634 |
| 9 | 6 and 8 | 302 |
| 10 | exp Meta analysis/ | 38,841 |
| 11 | ((meta adj analy$) or metaanalys$).tw. | 29,163 |
| 12 | (systematic adj (review$1 or overview$1)).tw. | 21,344 |
| 13 | 10 or 11 or 12 | 66,382 |
| 14 | cancerlit.ab. | 371 |
| 15 | cochrane.ab. | 10,826 |
| 16 | embase.ab. | 9,447 |
| 17 | (psychlit or psyclit).ab. | 484 |
| 18 | (psychinfo or psycinfo).ab. | 2,296 |
| 19 | (cinahl or cinhal).ab. | 2,989 |
| 20 | science citation index.ab. | 871 |
| 21 | bids.ab. | 214 |
| 22 | or/14-21 | 16,837 |
| 23 | reference lists.ab. | 3,572 |
| 24 | bibliograph$.ab. | 6,930 |
| 25 | hand-search$.ab. | 1,713 |
| 26 | manual search$.ab. | 1,206 |
| 27 | relevant journals.ab. | 313 |
| 28 | or/23-27 | 12,394 |
| 29 | data extraction.ab. | 6,440 |
| 30 | selection criteria.ab. | 8,261 |
| 31 | 29 or 30 | 14,222 |
| 32 | review.pt. | 1,038,101 |
| 33 | 31 and 32 | 8,195 |
| 34 | letter.pt. | 490,469 |
| 35 | editorial.pt. | 259,359 |
| 36 | animal/ | 55,517 |
| 37 | human/ | 7,099,174 |
| 38 | 36 not (36 and 37) | 38,607 |
| 39 | or/34-35,38 | 787,930 |
| 40 | 13 or 22 or 28 or 33 | 81,570 |
| 41 | 40 not 39 | 77,559 |
| **42** | **9 and 41** | 8 |

**Search strategy for ovidEMBASE**

| 1 | nurs*.mp. | 477,542 |
| --- | --- | --- |
| 2 | "nurs*".jn. | 124,158 |
| 3 | exp Korea/ | 14,731 |
| 4 | korea$.mp. | 22,562 |
| 5 | korea$.ia. | 94,914 |
| 6 | korea$.cp. | 22,620 |
| 7 | 1 or 2 | 515,645 |
| 8 | 3 or 4 or 5 or 6 | 108,378 |
| 9 | 7 and 8 | 2,446 |
| 10 | meta analy$.tw. | 29,075 |
| 11 | exp Meta-Analysis/ | 25,258 |
| 12 | metaanaly$.tw. | 940 |
| 13 | meta analysis.pt. | 25,258 |
| 14 | (systematic adj (review$1 or overview$1)).tw. | 21,880 |
| 15 | cochrane.ab. | 14,004 |
| 16 | cancerlit.ab. | 468 |
| 17 | embase.ab. | 11,499 |
| 18 | (psychlit or psyclit).ab. | 803 |
| 19 | (psychinfo or psycinfo).ab. | 3,488 |
| 20 | (cinahl or cinhal).ab. | 4,492 |
| 21 | science citation index.ab. | 1,141 |
| 22 | bids.ab. | 285 |
| 23 | or/15-22 | 21,572 |
| 24 | reference lists.ab. | 5,077 |
| 25 | bibliograph$.ab. | 8,379 |
| 26 | hand-search$.ab. | 2,389 |
| 27 | manual search$.ab. | 1,353 |
| 28 | relevant journals.ab. | 419 |
| 29 | or/24-28 | 15,824 |
| 30 | data extraction.ab. | 5,985 |
| 31 | selection criteria.ab. | 12,923 |
| 32 | 30 or 31 | 17,889 |
| 33 | review.pt. | 1,536,498 |
| 34 | 32 and 33 | 12,179 |
| 35 | letter.pt. | 682,887 |
| 36 | editorial.pt. | 258,334 |
| 37 | comment.pt. | 413,953 |
| 38 | animal/ | 4,593,791 |
| 39 | human/ | 11,294,941 |
| 40 | 38 not (38 and 39) | 3,412,328 |
| 41 | or/35-37,40 | 4,382,844 |
| 42 | exp Meta-Analysis as Topic/ | 10,376 |
| 43 | exp "Review"/ | 1,539,226 |
| 44 | "Review Literature as Topic"/ | 3,288 |
| 45 | 10 or 11 or 12 or 13 or 14 or 42 or 43 or 44 | 1,568,656 |
| 46 | 23 or 29 or 34 or 45 | 1,573,929 |
| 47 | 46 not 41 | 1,428,101 |
| **48** | **9 and 47** | 56 |
|  |  |  |

**Search strategy for KoreaMed**

| 1 | "systematic review" and nurs* | 4 |
| --- | --- | --- |
| 2 | "meta analysis" and nurs* | 38 |
| 3 | 1 or 2 | 42 |

**Search strategy for Korean Medical Databases**

| 1 | systematic review in title | 36 |
| --- | --- | --- |
| 2 | “metabunseok” in title | 43 |
| 3 | “meta bunseok” in title | 11 |
| 4 | “chegyejeok munheon” in title | 12 |
| 5 | or/1-4 | 102 |

**Search strategy for National Discovery for Science Leaders**

| 1 | "systematic review" in title or abstract(limited by Korean and Journals) | 22 |
| --- | --- | --- |
| 2 | “chegyejeok munheon gochal” in title or abstract (limited by Journals) | 4 |
| 3 | “meta bunseok” in title or abstract (limited by Journals) and “nursing” in searched results | 21 |
| 4 | or/1-3 | 47 |

**Search strategy for Korea Institute of Science and Technology Information**

| 1 | "systematic review" in title or abstract and “nursing” in searched results | 4 |
| --- | --- | --- |
| 2 | “chegyejeok munheon gochal” in title or abstract and “nursing” in searched results | 2 |
| 3 | "meta analysis" in title or abstract and “nursing” in searched results | 32 |
| 4 | “meta bunseok” in title or abstract and “nursing” in searched results | 21 |
| 5 | or/1-4 | 59 |

**Search strategy for Korean studies Information Service System**

| 1 | "systematic review in title or abstract and “nurs” in searched results | 0 |
| --- | --- | --- |
| 2 | "meta analysis” in title or abstract and “nurs” in searched results | 1 |
| 3 | “chegyejeok munheon” in title or abstract | 3 |
| 4 | "meta bunseok” in title or abstract and “nurs” in searched results | 20 |
| 5 | or/1-4 | 24 |
