# Supplementary material for: Quality assessment of systematic reviews or meta-analyses of nursing interventions conducted by Korean reviewers
Source: BMC Med Res Methodol. 2012 Aug 28;12:129. doi: 10.1186/1471-2288-12-129 (PMC3552770; doi:10.1186/1471-2288-12-129)
Supplement: Additional file 2 — Appendix 2. Reasons and a list of excluded reviews. [file 1471-2288-12-129-S2.doc]

**Appendix 2** Reasons and a list of excluded reviews

1. **Not investigated research question for nursing intervention (n = 7)**

Byeon YS, Oak JW: **Analysis of research trends in physiological variables in complementary and alternative therapy (CAT) in Korean nursing**. *J Korean Acad Fundament Nurs* 2006, **13**(2): 275-284. (Korean)

Kim BY, Lee CS: **A meta-analysis of variables related to suicidal ideation in adolescents**. *J Korean Acad Nurs* 2009, **39**(5): 651-661. (Korean)

Kim DS, Moon WH, Ahn SY, Oh HS, Kwon KH, Park MK, *et al*: **Meta-analysis of the research findings concerning functional relationships of explanatory variables to hope**. *J Korean Acad Nurs* 2004, **34**(5): 673-684. (Korean).

Kim YO: **The Effects of Hardiness : A meta-analysis of Korean nursing research findings**. *J Korean Acad Adult Nurs* 2005, **17**(5): 783-792. (Korean)

Lee HY: **Self-efficacy as a predictor of self-care in persons with diabetes mellitus: Meta- analysis**. *J Korean Acad Nurs* 1999, **29**(5): 1087-1102.

Park YJ, Lee SJ, Park ES, Ryu HS, Lee JW, Chang SO: **A meta-analysis of explanatory variables of health promotion behavior**. *J Korean Acad Nurs* 2000, **30**(4): 836-846. (Korean)

Yoo JS, Lee WH, Kim SS, Ko IS, Oh EG, Chu SH, *et al*: **Development of an Eye Care Protocol for ICU Patients**. *J Korean Acad Fundament Nurs* 2008, **15**(1): 34-44. (Korean)

1. **Narrative review (n = 9)**

Cho KH, Sung KW: **Study review of dying and death of the elderly**. *J Korean Acad Adult Nurs* 1997, **9**(3): 378-389.

Choi JH, Lee EO, Lee HY, Eun Y: **Analysis of the effects of Tai Chi on muscle strength and flexibility**. *J Muscle Joint Health* 2005, **12**(1): 69-80.

Eun Y, Lee HY, Choi JH, Lee EO: **Review of analysis of the effect of Tai Chi excercise on blood pressure and cardiopulmonary function**. *J Rheumatol Health* 2005, **12**(2): 132-142. (Korean)

Kim JH, Lim NY, So HY, Kang KS, Min HS, Park GH, *at al*: **An analysis on the research papers about exercise interventions to the stroke survivors**. *Korean J Rehabil Nurs* 2007, **10**(2): 116-124. (Korean)

Kim YO: **Analysis of nursing studies on hardiness published in Korea**. *J Korean Acad Adult Nurs* 2004, **16**(1): 27-36. (Korean)

Kim S, Song, M: **Comparison of non-pharmacological intervention programs for the Aged with hypertension in Korea and other countries**. *J Korean Geriatr Nurs* 2008, **10**(2): 152-163. (Korean)

Lee HY, Suh MJ, Lee EO, Eun Y, Choi JH: **Analysis of the effectiveness of Tai Chi exercise for improving balance**. *J Korean Acad Adult Nurs* 2004, **16**(3): 409-420. (Korean)

Oh J, Lee JH: **An analysis of nursing research related to the mother-child interaction in Korea**. *J Korean Acad Child Health Nurs* 2003, **9**(2): 149-161. (Korean)

Park YI, Susanna LC: **A review of self-monitoring of blood pressure for self-management of hypertension**. *J Korean Acad Soc Nurs Educ* 2007, **13**(1): 95-104. (Korean)
