# Supplementary material for: Quality assessment of systematic reviews or meta-analyses of nursing interventions conducted by Korean reviewers
Source: BMC Med Res Methodol. 2012 Aug 28;12:129. doi: 10.1186/1471-2288-12-129 (PMC3552770; doi:10.1186/1471-2288-12-129)
Supplement: Additional file 3 — Appendix 3. A list of included reviews. [file 1471-2288-12-129-S3.doc]

**Appendix 3** A list of included reviews

1. Lee EO, Song HH, Lee BS, Kim JH, Lee EH, Lee EJ, *et al*: **Effects of nursing interventions on anxiety and/or stress: a meta-analysis**. *J* *Korean Acad Community Health Nurs* 1992, **22**: 526-551. (Korean)
2. Lim SO, Hong EY: **An integrative review and meta-analysis of oncology nursing research: 1985-1997**. *J Korean Acad Nurs* 1996, **27**: 857-870. (Korean)
3. Kim NC, Song HH, Kim JO: **Effects of Nursing Interventions applied to surgery patients: a meta-analysis**. *J Korean Acad Adult Nurs* 1998, **10**: 523-534. (Korean)
4. Oh HS, Seo YO: **The comparison between the effects of integrated arthritis self-help programs and the effects of arthritis exercise programs through meta-analysis**. *J Korean Acad Nurs* 1998, **28**: 941-957. (Korean)
5. Kim EJ, Cho KM: **The Meta-analysis of Trends and Contents of Child Nursing Intervention Research**. *J Korean Acad Child Health Nurs* 2000, **6**: 119-131. (Korean)
6. Kim HS, Song HH, Choi SE: **A meta-analysis of effects of relaxation therapy on anxiety and blood pressure**. *J Korean Acad Nurs* 2000, **30**: 282-292. (Korean)
7. Park SH, Lee PS, Han KS: **Effect of relaxation therapy on anxiety through meta-analysis**. J *Korean Acad Psychiat Ment Health Nurs* 2001, **10**: 317-323. (Korean)
8. Park YJ, Oh KS, Lee SO, Oh KO, Kim JA, Kim HS, *et al*: **A Meta-Analysis of the Effects of the Supportive Nursing Intervention**. *J Korean Acad Child Health Nurs* 2001, **7**: 225-235. (Korean)
9. Oh WO, Suk MH: **A** **meta-analysis of the effects of imagery**. *J Korean Acad Nurs* 2002, **32**: 265-276. (Korean)
10. Kim EJ: **Meta-analysis on the Effects of Sensory Stimulation of Preterm Infants**. *J Korean Acad Child Health Nurs* 2003, **9**: 131-139. Korean.
11. Oh HS: **Meta-analysis on the effectiveness of interventions applied to preventing endotracheal suction-induced hypoxemia**. *J Korean Acad Nurs* 2003, **33**: 42-50. (Korean)
12. Oh HS: **Meta-analysis on the effectiveness of pulmonary rehabilitation program on exercise capacity/tolerance and general health status**. *J Korean Acad Nurs* 2003, **33**: 743-752. (Korean)
13. Lee KH, Park CJ. Kim MA, Park KM, Park JS, Shin YH, *et al*: **Effect of self regulation program on the hypertensive patient's self efficacy and self care through meta-analysis**. *J Korean Public Health Assoc* 2003, **29**: 269-274. (Korean)
14. Park EO: **A meta-analysis of the effects of smoking prevention programs in Korea**. *J Korean Acad Nurs* 2004, **34**: 1004-1013. (Korean)
15. Cha BK, Chang HK, Sohn JN: **A meta-analysis of the effects of a self-efficacy promoting program**. *J Korean Acad Nurs* 2004, **34**: 934-944. (Korean)
16. Yoo JS, Lee SJ: **A meta-analysis of the effects of exercise programs on glucose and lipid metabolism and cardiac function in patients with type Ⅱ diabetes mellitus**. *J Korean Acad Nurs* 2005, **35**: 546-554. (Korean)
17. Kim JH: **A meta-analysis of effects of job stress management intervention (SMIs)**. *J Korean Acad Nursing* 2007, **37**: 529-539. (Korean)
18. Choi H, Palmer MH, Park J: **Meta-analysis of pelvic floor muscle training: randomized controlled trials in incontinent women**. *Nurs Res* 2007, **56**: 226-234.
19. Kim Y, Park I, Park JS: **Meta-analysis of effects on adolescent smoking cessation programs in Korea**. *J Korean Acad Nurs* 2008, **38**: 204-216. (Korean)
20. Roh KH, Park HA: **A Meta-Analysis of the Effects of Aromatherapy on Psychological Variables in Nursing**. *J Korean Acad Community Health Nurs* 2009, **20**: 113-122. (Korean)
21. Yoo JS, Park JW, Lee SJ: **A Meta Analysis on the Effects of Exercise on Bone Mineral Density among Middle-aged and Older Women**. *J Korean Acad Community Health Nurs* 2009, **20**: 285-295. (Korean)
22. Jung DY, Lee JH, Lee SM: **A meta-analysis of fear of falling treatment programs for the elderly**. *West J Nurs Res* 2009, **31**: 6-16.
