# Supplementary material for: Quality assessment of systematic reviews or meta-analyses of nursing interventions conducted by Korean reviewers
Source: BMC Med Res Methodol. 2012 Aug 28;12:129. doi: 10.1186/1471-2288-12-129 (PMC3552770; doi:10.1186/1471-2288-12-129)
Supplement: Additional file 4 — Appendix 4. Characteristics of reviews included. [file 1471-2288-12-129-S4.doc]

**Appendix 4 Characteristics of reviews included**

| **Reference number**  **and first author** | | | **Publication year** | **Journal name** | **Patients/ Population** | **Intervention** | **Comparison** | **Outcomes** | **The number of studies included in meta-analysis** |
| --- | --- | --- | --- | --- | --- | --- | --- | --- | --- |
| 1 | Lee et al | 1992 | | Journal of Korean Academy of Nursing | healthy people and patients with diseases | nursing interventions on anxiety and stress(relaxation, education, touch/ supportive technique) | control/no program | biological indicators (blood pressure, pulse rate) | 64 experimental trials with randomized or nonequivalent control group pre-test/post-test design |
| 2 | Lim et al | 1996 | | Journal of Korean Academy of Nursing | various cancer patients | muscle relaxation, education, information, visiting intervention, holistic care, oral care, exercise program, ice bag, supportive care | control/ no program | control of nausea and vomiting, reduction of pain and anxiety, increase of food intake, improvement of quality of life, occurrence rate of stomatitis etc. | 25 quasi-experimental trials including pre-test/post-test design |
| 3 | Kim et al | 1998 | | Journal of Korean Academy of Adult Nursing | Surgery Patients | Nursing Interventions(relaxation, music therapy, heating, education) | self - control/ control groups | reduction of pain, muscle tension, and anxiety, stability of vital sign | 13 randomized or nonequivalent control groups in a pre-test/post-test design |
| 4 | Oh et al | 1998 | | Journal of Korean Academy of Nursing | arthritis patients | integrated arthritis self-help programs, exercise programs | self - control/ control groups | pain reduction, improvement of depression and functional status | 28 randomized or quasi-experimental trials including pre-test/post-test design |
| 5 | Kim et al | 2000 | | Journal of Korean Academy of Child Health Nursing | newborn, Infants, preschool, school age children | cognitive, movement, and social sensory interventions | self - control | change of mean effect size | 17 trials except primary experimental research |
| 6 | Kim et al | 2000 | | Journal of Korean Academy of Nursing | non-specific (healthy population, surgical patients, students' nurse, patients with coronary artery disease, adolescents) | various relaxation therapies | self - control | improvement of anxiety, change of blood pressure | 14 quasi-experimental trials including pre-test/post-test design |
| 7 | Park et al | 2001 | | Journal of Korean Academy of Psychiatric and Mental Health Nursing | depression, bipolar disorder, schizophrenia, surgical patients, students' nurse, fertile women, elderly residing in community facilities | various relaxation therapies | self - control | reduction of anxiety | 14 quasi-experimental trials including pre-test/post-test design |
| 8 | Park et al | 2001 | | Journal of Korean Academy of Child Health Nursing | diabetes, cancer patients, patients with tuberculosis, hypertension, inpatients, patients with back pain, hemodialysis patients, family with psychiatric disorders etc. | Supportive nursing interventions((1)supportive nursing behavior, (2)social support, and (3)combination of social support and supportive nursing behavior) | self - control/ control groups | role behavior compliance, anxiety, depression, health belief, knowledge about the disease, helplessness and stressful behavior response | 14 quasi-experimental trials including pre-test/post-test design |
| 9 | Oh et al | 2002 | | Journal of Korean Academy of Nursing | adolescents, psychiatric patients, pregnant women, hemodialysis patients, cancer patients, female university students, nurses, alcoholic patients | (behavioral and dynamic) Imagery interventions | self - control | change of stress, anxiety, depression, self-esteem, pulse rate, systolic blood pressure, quality of life, comfort, etc. | 15 quasi-experimental trials including pre-test/post-test design |
| 10 | Kim et al | 2003 | | Journal of Korean Academy of Child Health Nursing | Preterm Infants | sensory stimulations | self - control | increase of body weight, reduction of heart rate, change of saturated oxygen | 18 nonequivalent control groups in a pre-test/post-test design |
| 11 | Oh HS | 2003 | | Journal of the Korean Public Health Association | hypertensive patients | self-regulation program | self - control/ control groups | improvement of self efficacy and self care | 5 quasi-experimental trials including pre-test/post-test design |
| 12 | Oh HS | 2003 | | Journal of Korean Academy of Nursing | COPD patients | Pulmonary rehabilitation programs | control groups | improvement of exercise capacity/ tolerance and general health status | 17 randomized controlled trials |
| 13 | Lee et al | 2003 | | Journal of Korean Academy of Nursing | patients with various diseases, respiratory failure, coronary artery diseases | Intervention to prevent endotracheal suction-induced hypoxemia | self - control | occurrence rate of hypoxemia | 16 quasi-experimental trials including pre-test/post-test design |
| 14 | Park EO | 2004 | | Journal of Korean Academy of Nursing | students in elementary schools ~ high schools | smoking prevention programs | self - control/ no programs | knowledge, attitude, and behavioral changes | 20 quasi-experimental trials including pre-test/post-test design |
| 15 | Cha et al | 2004 | | Journal of Korean Academy of Nursing | patients with diabetes, hypertension, hemodialysis, cancer | self-efficacy promoting program(exercise, education, combination) | control/ no program | change of performance accomplishment, vicarious experience, verbal persuasion, and emotional arousal | 18 quasi-experimental trials |
| 16 | Yoo et al | 2005 | | Journal of Korean Academy of Nursing | type 2 diabetes | exercise programs | control groups/ no exercise | change of glucose, lipid metabolism, and cardiac function | 11 randomized controlled trials |
| 17 | Kim JH | 2007 | | Nursing Research | patients with urinary incontinence | pelvic floor muscle training | no treatment | incontinent episodes, urine leakage amount, and perceived severity of urine loss | 12 randomized controlled trials |
| 18 | Choi et al | 2007 | | Journal of Korean Academy of Nursing | workers in workplace | cognitive-behavioral intervention, relaxation techniques, exercise, organization-focused interventions | self - control/ control groups | reduction of workers' psycho-social and organizational Job stress | 46 randomized or nonequivalent control groups in a pre-test/post-test design |
| 19 | Kim et al | 2008 | | Journal of Korean Academy of Nursing | adolescent(middle and high school students) | smoking cessation programs(education based on Bandura’s social cognitive theory and cognitive-behavioral theory) | self - control/ control groups | change of smoking-knowledge, smoking-attitudes, and smoking-amount | 22 randomized or nonequivalent control groups in a pre-test/post-test design |
| 20 | Roh et al | 2009 | | Journal of Korean Academy of Community Health Nursing | healthy people and patients with diseases | Aromatherapy(massage, inhalation) | self - control | reduction of anxiety, depression, and stress | 31 quasi-experimental trials including pre-test/post-test design |
| 21 | Yoo et al | 2009 | | Journal of Korean Academy of Community Health Nursing | middle-aged and older women | exercise programs (weight bearing, strength, vibration, combination) | control groups/ no exercise | improvement of bone mineral density | 14 randomized controlled trials |
| 22 | Jung et al | 2009 | | Western Journal of Nursing Research | the elderly | Exercise, Education program, Hip protector | control/ no program | decrease of the fear of falling, increase of fall efficacy | 6 randomized controlled trials |
